# Supplementary material for: Recalibrating the epigenetic clock: implications for assessing biological age in the human cortex
Source: Brain. 2020 Oct 29;143(12):3763–75. doi: 10.1093/brain/awaa334 (PMC7805794; doi:10.1093/brain/awaa334)
Supplement: awaa334_Supplementary_Data [file awaa334_supplementary_data.pdf]

Supplementary data

Recalibrating the Epigenetic Clock: Implications for Assessing Biological Age in the Human Cortex

Gemma L Shireby[1], Jonathan P Davies[1], Paul T Francis[1,2], Joe Burrage[1], Emma M Walker[1], Grant W A Neilson[1], Aisha Dahir[1], Alan J Thomas[3], Seth Love[4], Rebecca G Smith[1], Katie Lunnon[1], Meena Kumari[5], Leonard C Schalkwyk[5], Kevin Morgan[6], Keeley Brookes[7], Eilis J Hannon[1*], Jonathan Mill[1*]

[1] University of Exeter Medical School, University of Exeter, Exeter, UK

[2] Wolfson Centre for Age-Related Diseases, King's College London, London, UK

[3]Institute of Neuroscience, Newcastle University, Newcastle Upon Tyne, UK

[4] Dementia Research Group, Institute of Clinical Neurosciences, School of Clinical Sciences, University of Bristol, Bristol, UK

[5]School of Biological Sciences, University of Essex, Colchester, UK

[6] Human Genetics Group, University of Nottingham, Nottingham, UK

[7]School of Science & Technology, Nottingham Trent University, Nottingham, UK

* These authors contributed equally.

Figure S1: Histograms showing the distribution of chronological age in the datasets used in the study. (**A**) The training dataset (n = 1,047 cortical samples); (**B**) the testing dataset (n = 350 cortical samples); (**C**) the independent test dataset (n = 1221 cortical samples) and (**D**) the whole blood dataset (n = 1175 whole blood samples).

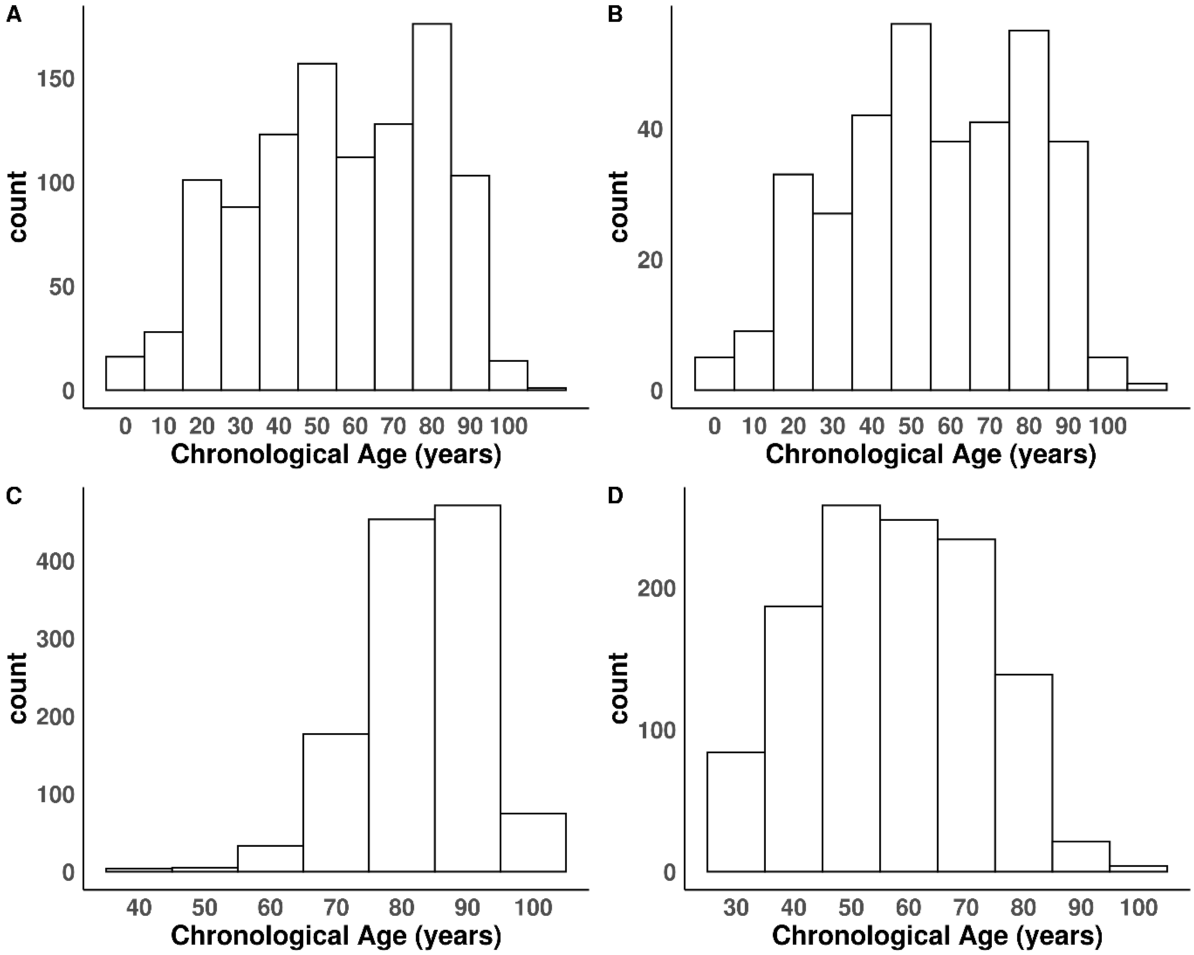

Figure S2: DNA methylation age has a logarithmic relationship with chronological age between the ages of 0-20 years. From 20 years onward there is a linear relationship with chronological age. The x-axis represents chronological age (years), the y-axis represents predicted age prior to applying the anti-transformation function, whereby age between 0-20 years is log transformed, and ages 20+ are transformed to account for this. Each point on the plot is a sample from the testing dataset (n = 350 cortical samples).

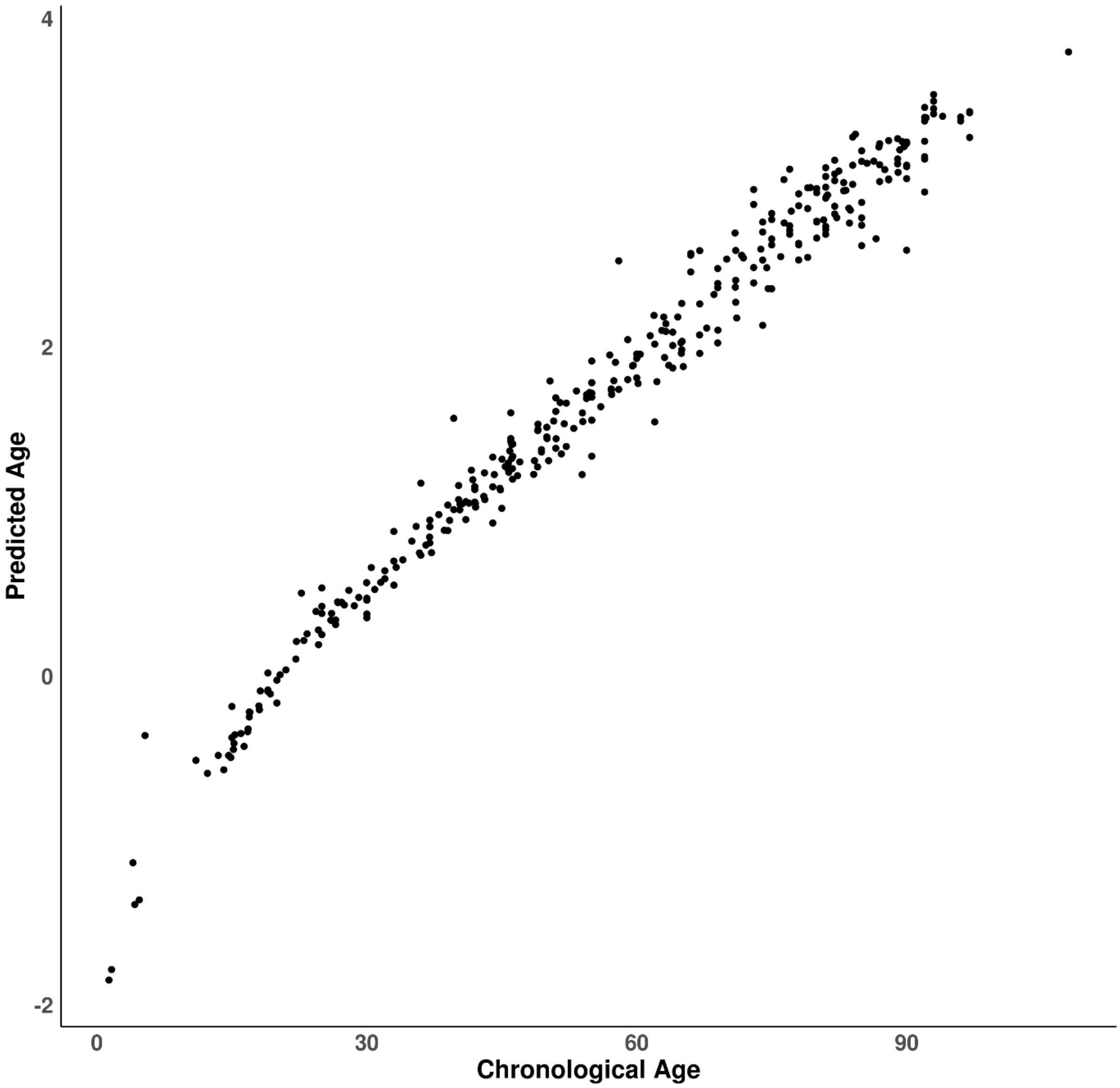

Figure S3: The cortical DNAm age clock has elevated accuracy in human cortex samples compared to existing DNAm clocks. Shown is the distribution of the error (DNA methylation age - chronological age) for each of the four DNA methylation age clocks in (**A**) the testing dataset (n = 350 cortical samples) and (**B**) the independent test dataset (n = 1,221 cortical samples). The ends of the boxes are the upper and lower quartiles of the errors, the horizontal line inside the box represents the median deviation and the two lines outside the boxes extend to the highest and lowest observations. Outliers are represented by points beyond these lines. The red horizontal line represents perfect prediction (zero error).

*DNAmClock$_{Cortical}$ = Cortical DNA methylation age Clock; DNAmClock$_{Multi}$ = Multi-tissue DNA methylation age clock; DNAmClock$_{Blood}$ = Blood DNA methylation age clock and DNAmClock$_{Pheno}$ = Pheno Age DNA methylation age clock.

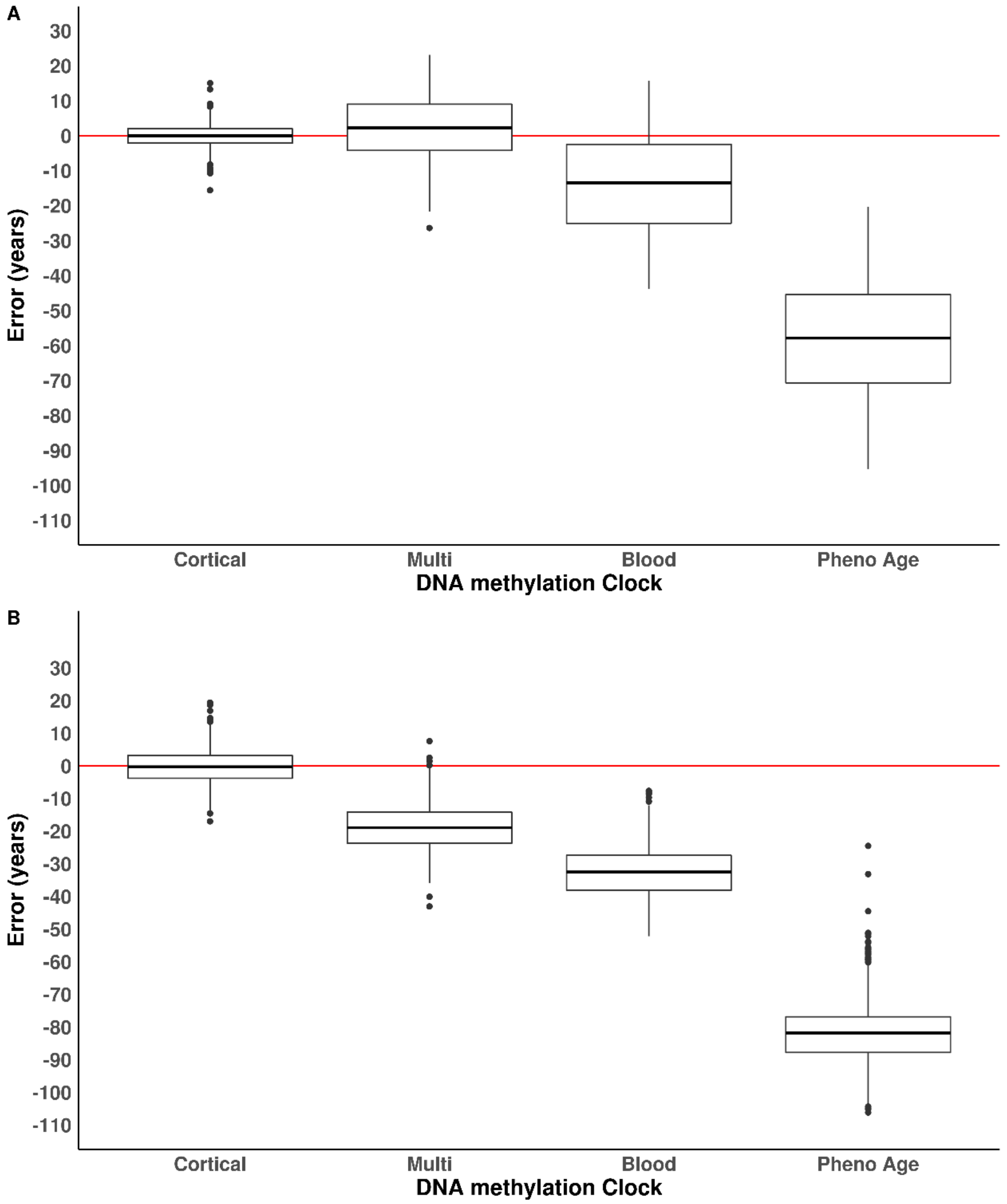

Figure S4: Bland-Altman plots highlighting enhanced performance of the cortical DNAm clock in human cortex tissue across the lifespan. Shown is the mean difference between actual chronological age and estimated DNAm ages derived in (**A**) the testing dataset (n= 350 cortical samples) and (**B**) the independent test dataset (n = 1221 cortical samples), where DNAm age derived using four DNA methylation age clocks: (**i**) our novel DNAmClock$_{\text{Cortical}}$; (**ii**) Horvath's DNAmClock$_{\text{Multi}}$; (**iii**) Zhang's DNAmClock$_{\text{Blood}}$ and (**iv**) Levine's DNAmClock$_{\text{Pheno}}$ The dashed horizontal lines in each case are the differences between actual and chronological age +/- 1.96 * standard deviation; for normally distributed difference due to error 5% points would lie outside these. The solid horizontal line represents where the difference would be zero.

*DNAmClock$_{\text{Cortical}}$ = Cortical DNA methylation age Clock; DNAmClock$_{\text{Multi}}$ = Multi-tissue DNA methylation age clock; DNAmClock$_{\text{Blood}}$ = Blood DNA methylation age clock and DNAmClock$_{\text{Pheno}}$ = Pheno Age DNA methylation age clock.

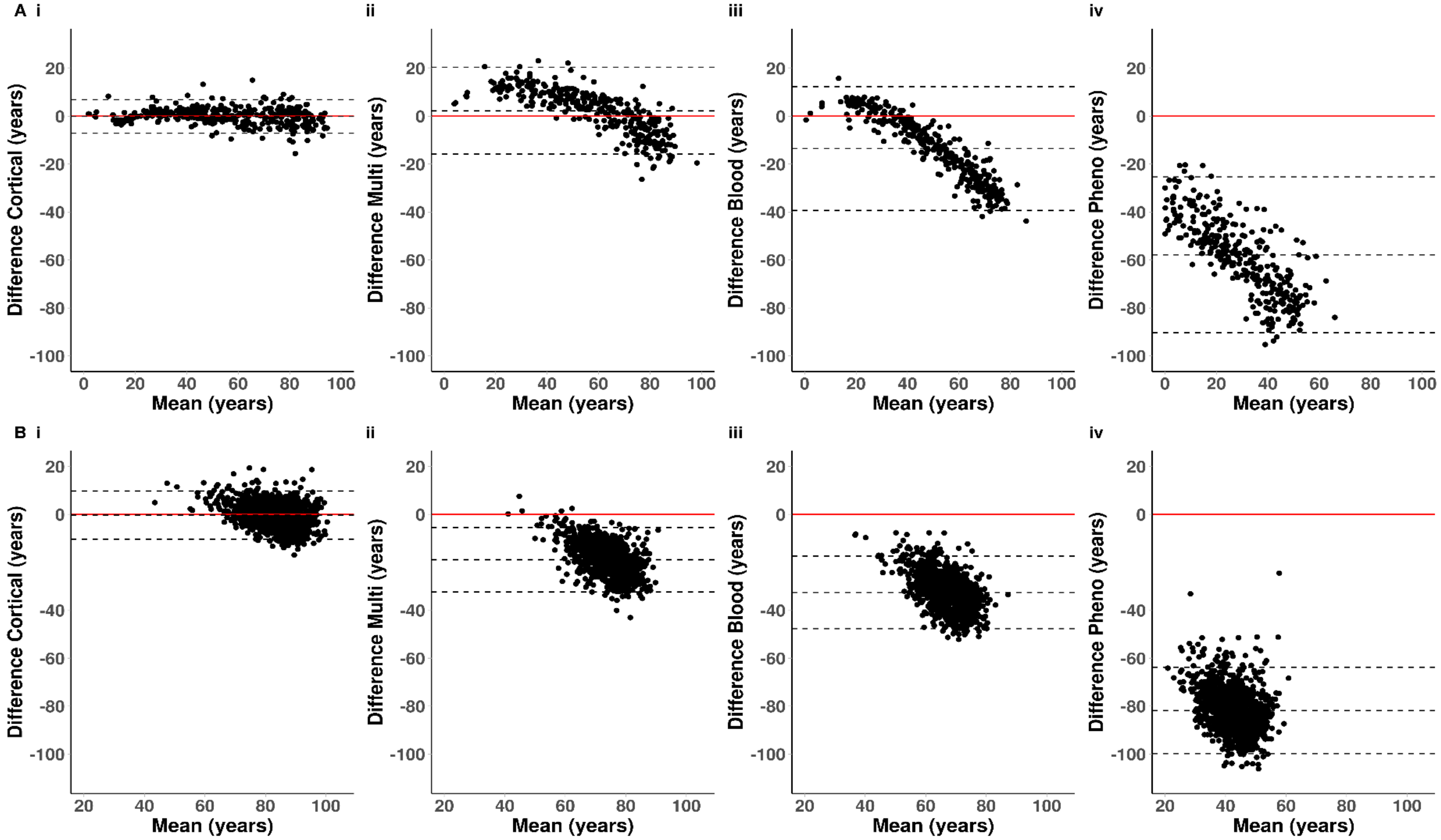

Figure S5: **The blood DNAm age clock has better accuracy in human whole blood samples compared to non-tissue specific DNAm clocks.** Distribution of the error in years (DNA methylation age - chronological age) comparing four DNA methylation age clocks: our novel $DNAmClock_{Cortical}$, the $DNAmClock_{Multi}$, the $DNAmClock_{Blood}$ and the $DNAmClock_{Pheno}$ in the **whole blood dataset** (n = 1175). The ends of the boxes are the upper and lower quartiles of the errors, the horizontal line inside the box represents the mean absolute deviation and the two lines outside the boxes extend to the highest and lowest observations. Outliers are represented by points beyond these lines.

*$DNAmClock_{Cortical}$ = Cortical DNA methylation age Clock; $DNAmClock_{Multi}$ = Multi-tissue DNA methylation age clock; $DNAmClock_{Blood}$ = Blood DNA methylation age clock and $DNAmClock_{Pheno}$ = Pheno Age DNA methylation age clock.

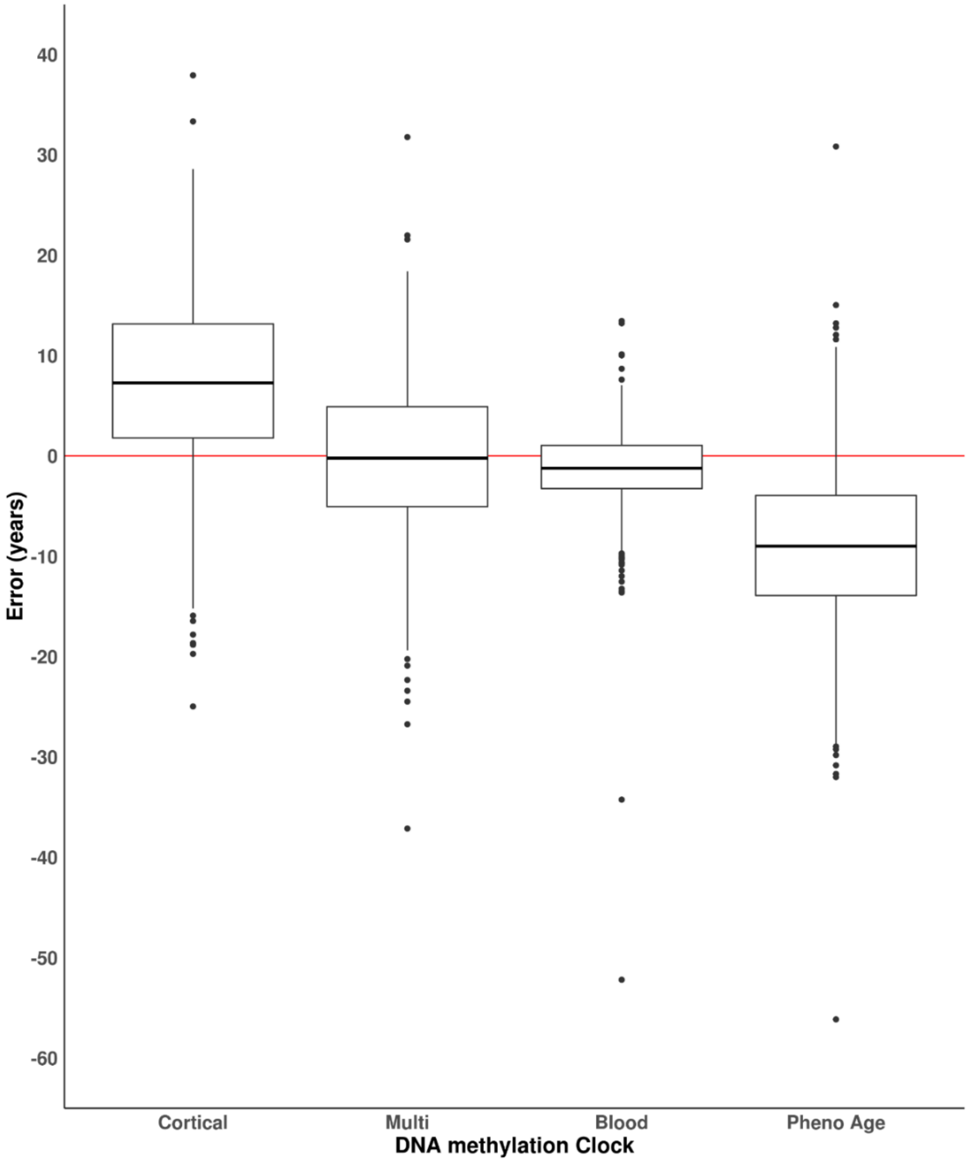

Figure S6: **Bland-Altman plots highlighting elevated performance of blood based DNAm clocks in whole blood samples.** Mean-difference (Bland-Altman) plots showing the difference between DNA methylation age estimates against chronological age using (**A**) our novel $\text{DNAmClock}_{\text{Cortical}}$, (**B**) the $\text{DNAmClock}_{\text{Multi}}$, (**C**) the $\text{DNAmClock}_{\text{Blood}}$ and (**D**) the $\text{DNAmClock}_{\text{Pheno}}$ in the whole blood cohort (n = 1175). The dashed horizontal lines in each case are the differences between actual and chronological age +/- 1.96 * standard deviation; for normally distributed difference due to error 5% points would lie outside these. The solid horizontal line represents where the difference would be zero.

*$\text{DNAmClock}_{\text{Cortical}}$ = Cortical DNA methylation age Clock; $\text{DNAmClock}_{\text{Multi}}$ = Multi-tissue DNA methylation age clock; $\text{DNAmClock}_{\text{Blood}}$ = Blood DNA methylation age clock and $\text{DNAmClock}_{\text{Pheno}}$ = Pheno Age DNA methylation age clock.

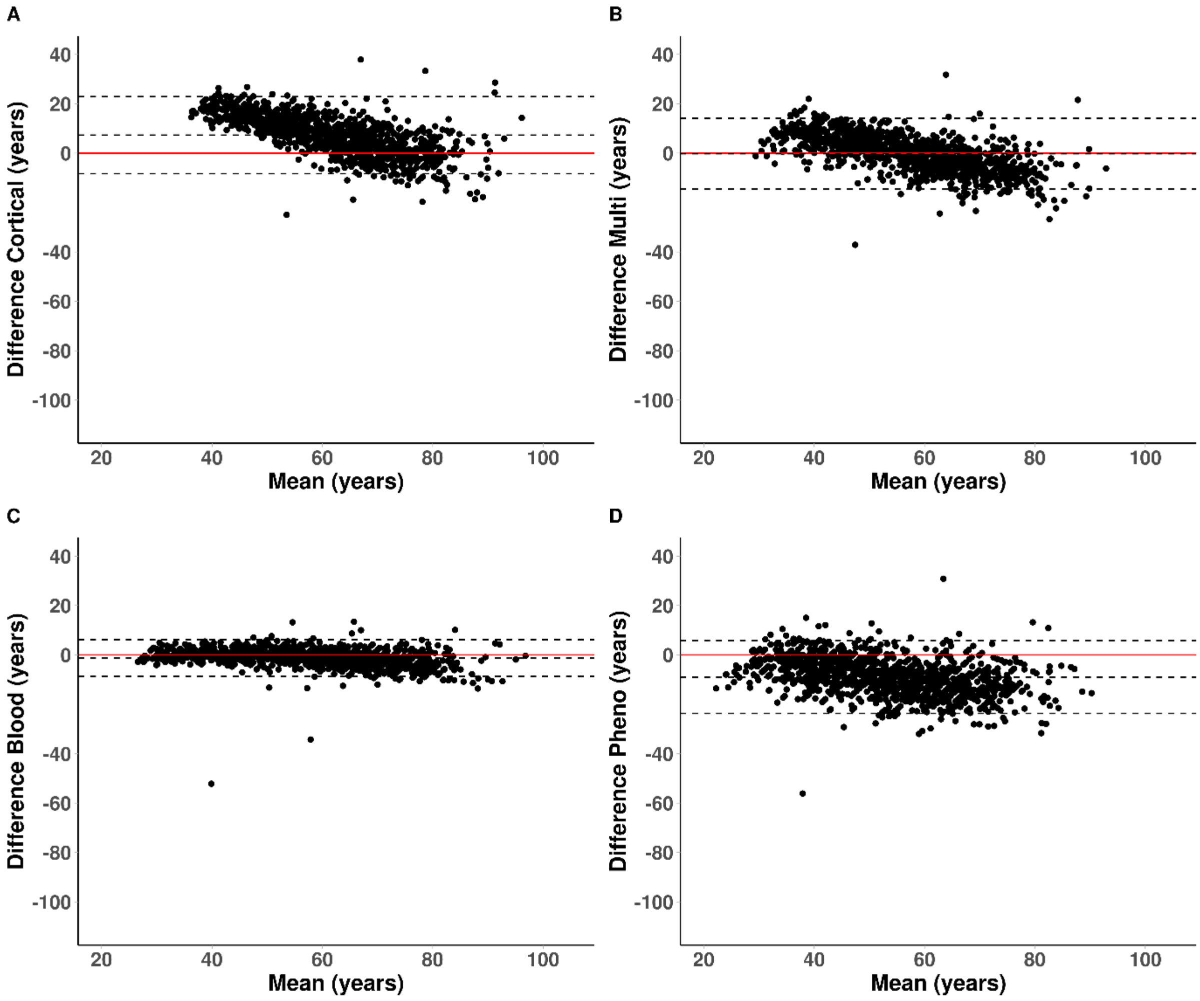

Figure S7: The blood-based DNA methylation clock performs best in data derived from whole blood samples. (**A**) Shown is a comparison of DNA methylation age estimates against chronological age in samples >55 years old from a large whole blood dataset (n = 646), where DNAm age is derived using four DNA methylation age clocks: (**i**) our novel $DNAmClock_{Cortical}$; (**ii**) Horvath's $DNAmClock_{Multi}$; (**iii**) Zhang's $DNAmClock_{Blood}$ and (**iv**) Levine's $DNAmClock_{Pheno}$. The x-axis represents chronological age (years), the y-axis represents predicted age (years). Each point on the plot represents an individual in the whole blood dataset. Our novel clock does not predict as well as in the cortex, although it has a similar predictive ability to Horvath's clock. The distribution of the error (DNA methylation age - chronological age) is presented in (**B**) for each decile for each of the four DNA methylation clocks. Deciles were calculated by assigning chronological age into ten bins and are represented along the x-axis by the numbers one to ten, followed by brackets which display the age range included in each decile. The ends of the boxes are the upper and lower quartiles of the errors, the horizontal line inside the box represents the median deviation and the two lines outside the boxes extend to the highest and lowest observations. Outliers are represented by points beyond these lines. The red horizontal line represents perfect prediction (zero error).

*$DNAmClock_{Cortical}$ = Cortical DNA methylation age Clock; $DNAmClock_{Multi}$ = Multi-tissue DNA methylation age clock; $DNAmClock_{Blood}$ = Blood DNA methylation age clock and $DNAmClock_{Pheno}$ = Pheno Age DNA methylation age clock.

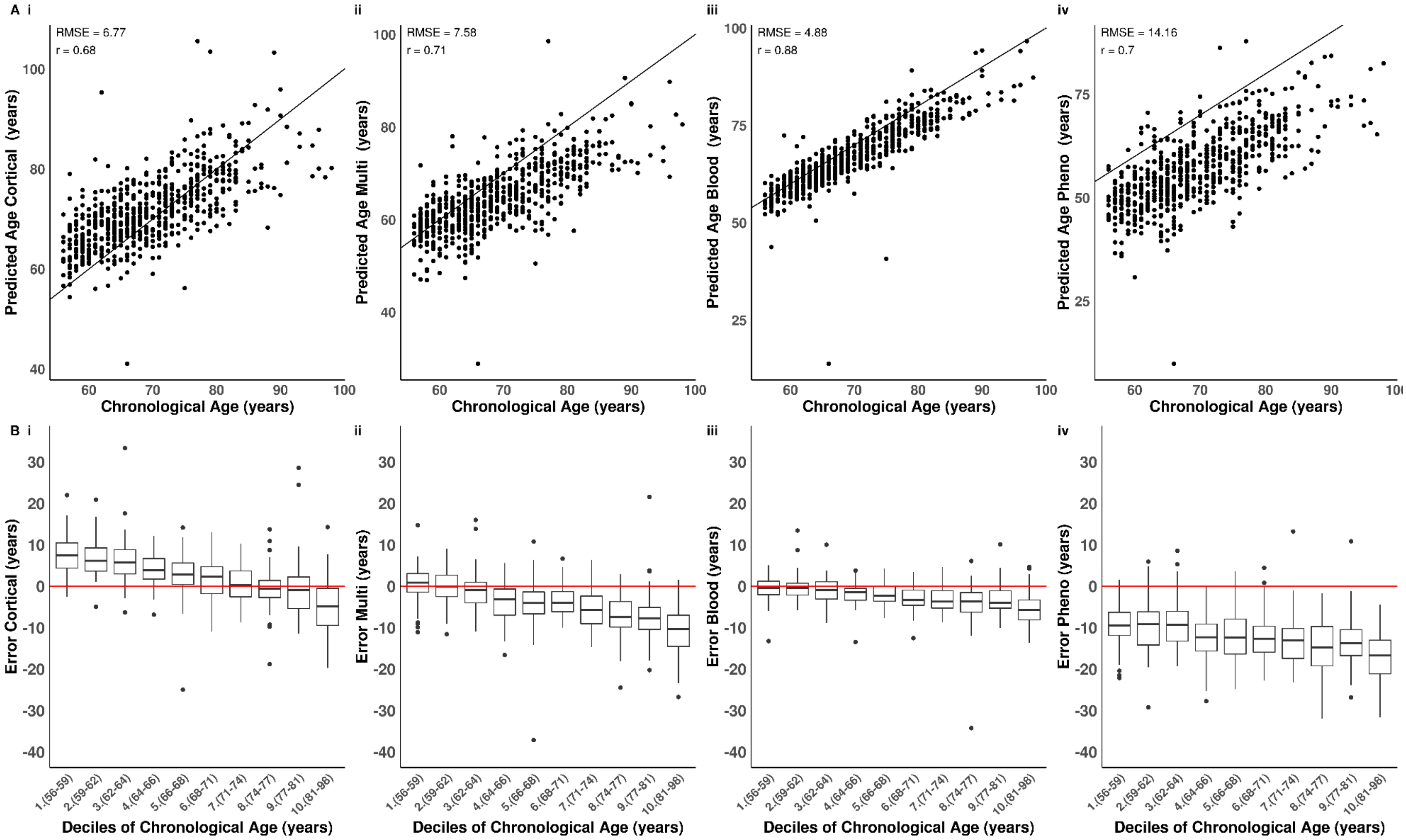

A i
RMSE = 6.77
r = 0.68
Predicted Age Cortical (years)
Chronological Age (years)
ii
RMSE = 7.58
r = 0.71
Predicted Age Multi (years)
Chronological Age (years)
iii
RMSE = 4.88
r = 0.88
Predicted Age Blood (years)
Chronological Age (years)
iv
RMSE = 14.16
r = 0.7
Predicted Age Pheno (years)
Chronological Age (years)
B i
Error Cortical (years)
Deciles of Chronological Age (years)
ii
Error Multi (years)
Deciles of Chronological Age (years)
iii
Error Blood (years)
Deciles of Chronological Age (years)
iv
Error Pheno (years)
Deciles of Chronological Age (years)
1.(56-59)
2.(59-62)
3.(62-64)
4.(64-66)
5.(66-68)
6.(68-71)
7.(71-74)
8.(74-77)
9.(77-81)
10.(81-98)

Supplementary Table S1: Sample characteristics of the training (cortex), testing (cortex), independent test (cortex) and whole blood datasets used in the development and evaluation of our novel cortical DNA methylation clock including the number of samples from each brain region.

Dataset	Tissue Type	Brain regions (n)	N	Age (years)				Sex (n)		Illumina methylation array	Reference	GEO Accession number
				Mean	Median	Range	SD	Female	Male			
**Training**	Cortical	**BA[9,11,17,25,41]** (284); **EC**(59); **HIP** (31); **PFC** (513); **STG** (83); **STR** (77)	1047	56.53	57	1.34-108	24.13	362	685	450K	(Jaffe et al., 2016; De Jager et al., 2014; Lunnon et al., 2014; Pidsley et al., 2014; Smith et al., 2018, 2019; Wong et al., 2019)	GSE74193; GSE59685; GSE80970; GPL13534; GSE43414;
**Testing**	Cortical	**BA[9,11,17,25,41]** (97); **EC**(18); **HIP** (11); **PFC** (167); **STG** (37); **STR** (20)	350	55.87	56	1.34-108	24.25	144	206	450K	(Jaffe et al., 2016; De Jager et al., 2014; Lunnon et al., 2014; Pidsley et al., 2014; Smith et al., 2018, 2019; Wong et al., 2019)	GSE74193; GSE59685; GSE80970; GPL13534; GSE43414;
**BDR**	Cortical	**PFC** (612) **OCC** (609)	1221	83.49	84	41-104	9.10	577	644	EPIC	-	
**Understanding Society**	Whole Blood	-	1175	57.96	59	28-98	14.97	686	489	EPIC	Hannon et al. (2018)	

* BA = Brodmann area; EC = Entorhinal cortex; HIP = Hippocampus; PFC = Prefrontal cortex; OCC = Occipital lobe; STG = Superior temporal gyrus; STR = Striatum; SD = Standard deviation; GEO = Gene Expression Omnibus

Supplementary Table S2: Age statistics for cohorts included in the training (cortex) and testing (cortex) datasets used in the development and evaluation of our novel cortical DNA methylation clock including the number of samples from each cohort.

Cohort / Brain Bank	N	Age (years)						Sex (n)	
		1st quartile	Median	Mean	3rd quartile	Range	SD	Female	Male
**Edinburgh**	45	37	42	43.22	51	18-69	13.45	7	38
**Harvard**	54	16.25	26.5	28.39	39	2-60	15.79	11	43
**Lieber Institute**	566	28.42	45.14	42.45	55.42	1.34-96.98	18.6	197	369
**LBB2**	27	67.5	80	78.56	88.5	58-99	11.32	14	13
**Maudsley**	282	64	79	73.15	86	25-105	17.06	123	159
**Montreal**	137	30	41	44.47	57	18-90	18.28	28	109
**MS**	125	76	82	82.89	89	70-108	7.88	64	61
**NICHD**	18	15	21.5	26.61	42.75	4-67	18.55	0	18
**Oxford**	29	62	64	62.38	68	41-71	8.51	9	20
**ROSMAP**	114	77.32	81.82	81.45	86.18	65.99-89.73	5.45	53	61

* LBB2 = London Brain Bank 2; MS = Mount Sinai; NICHD = National Institute of Child Health and Human Development; ROSMAP = Religious Orders Study and Rush Memory and Aging Project; SD = Standard Deviation

Supplementary Table S3: The Cortical Clock DNA methylation probes and their coefficients. Using elastic net regression we identified 347 DNA methylation sites which in combination optimally predict age in the human cortex. The sum of DNAm levels at these sites weighted by their regression coefficients provides the Cortical Clock DNA methylation age estimate. These coefficient values relate to a transformed version of age and therefore the linear combination of CpGs needs to be suitably transformed.

Illumina Probe ID	Coefficient
(Intercept)	0.57768257
cg00059225	0.24595921
cg00088042	0.18271556
cg00252534	0.16822336
cg00297950	0.14175773
cg00384539	0.17820188
cg00491255	0.14450779
cg00521255	-0.2443627
cg00648582	-0.0391462
cg00771642	0.12772573
cg00924265	-0.0685163
cg00935119	-0.038119
cg00940577	-0.0612173
cg01091514	-0.0322298
cg01122755	0.06571091
cg01162920	-0.1973567
cg01194538	-0.1516094
cg01264729	-0.1524237
cg01311102	-0.1092163
cg01529637	0.02680964
cg01532168	0.33465593
cg01616394	-0.0187101
cg01639032	0.5150527
cg01641432	-0.0013768
cg01655150	-0.3276135
cg01745370	-0.0058643
cg01899542	-0.0509626
cg02046143	-0.0077791
cg02047661	-0.0171169
cg02357838	0.23797342
cg02361903	-0.5452234
cg02397497	0.07620684
cg02448244	0.03955555
cg02504211	0.13446075
cg02524236	-0.058474
cg02546818	0.01616236
cg02583546	-0.0359673
cg02795151	-0.0056271
cg02983424	0.05696411
cg03001484	-0.0258987
cg03025830	0.13631721
cg03040821	-0.0574853
cg03048488	-0.1256683
cg03591753	-0.4520824
cg03594801	-0.0691302
cg03613618	-0.1393547

cg03633073	0.24465057
cg03639603	0.07133576
cg03710354	0.00012886
cg03717616	-0.1918336
cg03854598	0.19543214
cg03864215	-0.3501439
cg04031134	0.02070123
cg04036898	-0.0001322
cg04055913	-0.0657139
cg04060163	-0.0111752
cg04078896	-0.0120007
cg04235075	0.20720766
cg04315771	-0.052222
cg04370442	0.07671797
cg04432319	0.37983386
cg04604946	-0.1166149
cg04684267	0.56103948
cg04686264	0.09996322
cg04704414	-0.0249742
cg04834794	0.86355274
cg04880546	0.03041421
cg04913265	0.05506598
cg05006304	-0.099641
cg05030953	-0.012153
cg05149386	-0.1053181
cg05213896	0.01844346
cg05280698	0.25970942
cg05333146	0.19252122
cg05362168	-0.0134173
cg05396044	0.9444237
cg05404236	0.09491394
cg05444541	-0.0118706
cg05634040	-0.0761376
cg05724492	0.03479139
cg05756780	0.06888371
cg05785488	0.58125152
cg05795849	-0.0377685
cg05839636	-0.0709078
cg06069616	-0.0128466
cg06144905	0.05548133
cg06236737	-0.4656742
cg06385324	0.08016557
cg06570818	-0.0040639
cg06645033	0.23463392
cg06648759	0.40674287
cg06711656	0.15663601
cg06751446	0.08353432
cg06852461	-0.0063935
cg07011538	-0.0337366
cg07120479	-0.4293158
cg07181374	-0.1310245
cg07197480	0.04978597
cg07461572	-0.0014446
cg07544187	0.19887884
cg07547549	0.01343572

cg07550554	0.2486126
cg07581257	-0.1444611
cg07806886	0.01131
cg08097417	0.55988863
cg08193650	0.07922242
cg08370996	0.124238
cg08594681	-0.0976416
cg08606497	-0.0136992
cg08708711	0.03540909
cg08727193	0.11187629
cg08786136	0.13167062
cg08952475	-0.0018342
cg08995871	0.0004663
cg09058748	0.00609294
cg09124496	-0.0726601
cg09178970	-0.0244848
cg09254686	-0.2087676
cg09363564	-0.1487783
cg09372546	-0.018437
cg09407967	0.07651973
cg09468836	0.02747043
cg09664474	-0.711323
cg09731141	-0.002565
cg09810078	0.03982331
cg09888620	0.22004768
cg09906752	-0.015249
cg09935271	-0.0195523
cg10027085	-0.0038364
cg10104252	-0.0272235
cg10225362	0.04192172
cg10232140	-0.0178474
cg10359006	-0.1063366
cg10378521	-0.2043615
cg10389229	0.00641507
cg10460946	-0.2376023
cg10501210	-0.305486
cg10574566	-0.0829055
cg10790698	-0.1026348
cg10851350	-0.0098493
cg10904972	-0.0784357
cg10924085	-0.0343679
cg10961323	0.0117429
cg11018337	0.10227319
cg11071401	0.00301298
cg11120115	-0.1945644
cg11397957	-0.1287726
cg11462165	0.1817344
cg11565355	0.11512885
cg11603443	0.11675982
cg11719412	-0.0717141
cg12024906	0.05166947
cg12100751	0.08174288
cg12175729	0.07132377
cg12423733	-0.0653703
cg12591491	0.24421376

cg12597389	0.5583175
cg12637942	0.17055797
cg12950231	-0.0656435
cg12978800	-0.1818211
cg13099374	-0.0473964
cg13259357	0.03688023
cg13298199	-0.0971264
cg13308350	-0.2068517
cg13327545	0.17202187
cg13477806	0.03348578
cg13718185	-0.0024661
cg13733708	-0.0027441
cg13744194	0.09010979
cg13755546	0.16217758
cg13806267	0.02601212
cg13848598	0.31150974
cg14189141	0.14486526
cg14242024	-0.0891087
cg14343652	-0.4916114
cg14489570	-0.0668353
cg14507337	0.04659403
cg14577472	0.0484974
cg14611683	0.05482077
cg14655122	0.2417095
cg14704921	0.21510624
cg14759277	0.11132052
cg14871932	0.10868066
cg15001747	0.43298364
cg15022387	0.04441422
cg15341124	0.90243633
cg15393490	-0.8410718
cg15410236	0.21389648
cg15540623	-0.2615207
cg15593298	0.33773506
cg15638207	-0.6916681
cg15665342	-0.0605596
cg15718663	0.11706067
cg15925792	-0.307196
cg15927455	-0.3584996
cg15974867	0.02314957
cg15988970	-0.0064278
cg16121765	-0.0123589
cg16142349	-0.2568507
cg16148593	-0.0981528
cg16206504	-0.0083124
cg16339238	0.00143445
cg16340422	-0.0292647
cg16359034	-0.0461923
cg16408865	-0.1234816
cg16604975	0.01666265
cg16643261	-0.1185516
cg16703882	0.09662046
cg16865965	-0.0417045
cg16867657	0.19738643
cg16909962	0.26618242

cg17040471	0.01800027
cg17086398	-0.2146144
cg17117277	0.41446544
cg17341113	1.44006579
cg17411994	-0.0762994
cg17435266	0.06161905
cg17523253	-0.0670554
cg17592231	0.68799031
cg17640485	-0.3129539
cg17693222	0.00318175
cg18077971	0.0867302
cg18125865	0.65840184
cg18247055	0.01999414
cg18279094	0.35656711
cg18427787	-0.0441448
cg18449120	0.00500354
cg18468088	0.22981384
cg18480946	0.00979905
cg18504218	-0.008073
cg18514820	0.22286731
cg18540328	0.02261323
cg18549036	0.00040885
cg18584803	-0.1976921
cg18604199	-0.0521892
cg18626323	-0.0303841
cg19028706	-0.0901704
cg19056004	-0.043383
cg19142026	0.24687437
cg19230755	0.00276837
cg19242851	-0.1708169
cg19399220	0.23167516
cg19416570	0.21729811
cg19699893	0.13548726
cg19724470	-0.0496928
cg19802138	0.01144858
cg19807317	-0.0546721
cg19955173	-0.0675901
cg20185454	0.18559061
cg20198242	-0.0058134
cg20429250	0.02217301
cg20495179	-0.3422078
cg20516262	-0.0036741
cg20583430	0.04465635
cg20594982	0.20060143
cg20603637	0.04123459
cg20627572	-0.037941
cg20697204	0.04133236
cg20747577	-0.0067257
cg20773033	-0.205063
cg20818778	0.03589532
cg21010435	0.00127567
cg21052766	-0.0232065
cg21186299	0.05465318
cg21218687	0.12544553
cg21415530	0.00975912

cg21581504	0.10314205
cg21801378	0.3019194
cg21826815	0.14908739
cg21851534	-0.0365721
cg21865150	-0.0024381
cg21967909	0.13778356
cg22007809	-0.0919512
cg22131013	-0.0213741
cg22285878	0.49092431
cg22320999	-0.0141227
cg22344793	0.00901416
cg22363327	0.0637605
cg22531668	0.03617343
cg22661556	0.10352047
cg22714290	-0.0507599
cg22884541	0.14417822
cg22900415	0.04351403
cg23051272	-0.0698149
cg23091758	0.11685302
cg23124451	-0.3676633
cg23163283	-0.1166281
cg23166770	-0.4055405
cg23174607	0.47022718
cg23201812	-0.0043506
cg23352942	-0.0709361
cg23474190	-0.0992123
cg23606718	0.25215709
cg23636833	0.05409979
cg23661344	-0.0953841
cg23662138	-0.1453696
cg23684204	0.36656376
cg23813012	0.09645841
cg23896431	0.10834819
cg23939875	-0.0027469
cg23995914	0.13059076
cg24085039	-0.0266476
cg24231804	-0.0610665
cg24287218	-0.057864
cg24346776	0.03688965
cg24377285	-0.0034626
cg24420164	0.01149875
cg24483655	-0.1180724
cg24567591	0.00663023
cg24715767	0.03440595
cg24718465	0.09807228
cg24933925	-0.0626709
cg24990808	-0.0417352
cg25007705	-0.2476066
cg25018458	0.51960078
cg25090514	0.5000666
cg25114611	-0.1558847
cg25201359	-0.3919139
cg25459323	-0.0912804
cg25922329	-0.0449031
cg26092675	0.09283213

cg26115633	0.27801401
cg26161329	0.64983683
cg26242531	-0.3438033
cg26306636	0.67247185
cg26332630	-0.0217055
cg26377000	0.06837371
cg26384036	0.08320489
cg26472036	-0.2159495
cg26490949	0.39372896
cg26542283	0.06460673
cg26645401	0.23806474
cg26660754	-0.3879276
cg26726230	0.08107185
cg26782108	0.09393526
cg26856080	0.25484991
cg26885220	0.15739762
cg26952697	-0.0575663
cg26952796	-0.0916588
cg27013696	0.18890756
cg27043838	0.00732047
cg27134767	-0.1281932
cg27230784	0.11078553
cg27388680	-0.1024366
cg27414487	-0.0433871
cg27529628	0.01778693
ch.2.1904845F	-0.1201514
ch.2.71774667F	-0.2699354

Supplementary Table S4: Overlap between the probes which collectively make up the Cortical DNA methylation clock and three other DNA methylation age clocks: Horvath's Multi tissue DNA methylation Clock, Zhang's Blood DNA methylation Clock and Levine's Pheno Age DNA methylation Clock.

Overlap Horvath Multi Tissue	Overlap Zhang Blood	Overlap Levine Pheno Age
cg06144905	cg02046143	cg06144905
cg08370996	cg03025830	cg19724470
cg19724470	cg04604946	cg21801378
cg21801378	cg04684267	cg23124451
cg23124451	cg06648759	cg25459323
	cg07547549	
	cg08097417	
	cg09935271	
	cg15393490	
	cg16867657	
	cg18468088	
	cg21186299	
	cg23174607	
	cg23606718	
	cg23995914	

Supplementary Table S5: The relationship between DNAm age and technical and biological variables. DNAm age was estimated using our novel cortical clock in the independent test dataset (n=1,221). DNAm age was regressed against biological and technical variables (cell proportions, sex, post-mortem interval and batch).

	Beta	SE	P
**Cell proportions**	-8.72	0.67	9.57E-36
**Sex**	0.60	0.28	0.03
**PMI**	-3.90E-03	0.01	0.54
**Batch**	-0.03	0.03	0.38

*DNAm = DNA methylation; PMI = Post-mortem interval
